# Modelling Human Locomotion to Inform Exercise Prescription for Osteoporosis

**DOI:** 10.1007/s11914-020-00592-5

**Published:** 2020-04-25

**Authors:** Saulo Martelli, Belinda Beck, David Saxby, David Lloyd, Peter Pivonka, Mark Taylor

**Affiliations:** 1 Medical Device Research Institute, College of Science and Engineering, Flinders University, Tonsley, SA 5042 Australia; 2 School of Allied Health Sciences, Griffith University, Gold Coast, QLD Australia; 3 Griffith Centre of Biomedical and Rehabilitation Engineering (GCORE), Menzies Health Institute Queensland, Griffith University, Gold Coast, QLD Australia; 4 School of Chemistry, Physics and Mechanical Engineering Queensland University of Technology, Brisbane, Australia

**Keywords:** Hip strain, Physical exercise, Osteoporosis, Bone strength

## Abstract

**Purpose of Review:**

We review the literature on hip fracture mechanics and models of hip strain during exercise to postulate the exercise regimen for best promoting hip strength.

**Recent Findings:**

The superior neck is a common location for hip fracture and a relevant exercise target for osteoporosis. Current modelling studies showed that fast walking and stair ambulation, but not necessarily running, optimally load the femoral neck and therefore theoretically would mitigate the natural age-related bone decline, being easily integrated into routine daily activity. High intensity jumps and hopping have been shown to promote anabolic response by inducing high strain in the superior anterior neck. Multidirectional exercises may cause beneficial non-habitual strain patterns across the entire femoral neck. Resistance knee flexion and hip extension exercises can induce high strain in the superior neck when performed using maximal resistance loadings in the average population.

**Summary:**

Exercise can stimulate an anabolic response of the femoral neck either by causing higher than normal bone strain over the entire hip region or by causing bending of the neck and localized strain in the superior cortex. Digital technologies have enabled studying interdependences between anatomy, bone distribution, exercise, strain and metabolism and may soon enable personalized prescription of exercise for optimal hip strength.

**Electronic supplementary material:**

The online version of this article (10.1007/s11914-020-00592-5) contains supplementary material, which is available to authorized users.

## Introduction

Reduced bone mass and compromised bone strength in osteoporosis are a major socio-economic burden. The health care cost for treating low and minimal energy fragility fractures was $2.8 billion in Australia in 2012, and it is expected to rise to $3.8 billion by 2022 due to population growth and ageing [1]; this figure is common to most regions of the developed world [2]. Physical exercise has long been proposed as a means for reducing the risk of fragility fracture by promoting bone accrual and improving motor function to reduce fall risk. However, the modest and variable bone response to exercise in adults complicates prescribing the type and amount of exercise for optimal bone health [3]. Modern digital technology can complement exercise prescription for osteoporosis by providing information about skeletal mechanics during locomotion.

A recent position statement endorsed by Exercise and Sport Science Australia (ESSA) summarized current research findings into specific guidelines for the prevention and management of osteoporosis through exercise [3]. In older adults, regular impact and high intensity resistance exercises may reduce the likelihood of both falling and fracture by enhancing musculoskeletal function and promoting modest bone accrual [3–7], yet the effect has been observed to be highly variable [8]. For example, in women over 58 years of age, percent change (mean ± SD) in femoral neck bone mineral density (BMD) in response to an 8-month training program including high intensity resistance and weight-bearing activities was 0.3 ± 3.0% compared with − 2.0 ± 3.0% in sedentary controls [6], thereby showing a variance larger than the mean effect (CV > 100%). An understanding of the patterns of bone strain during different types of exercise together with longitudinal information on BMD may help optimize exercise programs to improve bone strength and mitigate the incidence of fragility fractures.

Digital technology can complement randomized controlled trial (RCT) designs by combining principles of mechanics, muscle and bone physiology with anatomical and functional measurements in participants into physics-based models, which can provide information relating to musculoskeletal loading during exercise otherwise not accessible from direct human measures. For example, physics-based models are increasingly used in a variety of clinical contexts of increasing breath [9] such as studies of musculoskeletal function in healthy adults [10••], obese children [11] and joint replacements [12]. Models provided muscle and joint forces [10••, 13, 14] and bone strain [15, 16] and improved prediction of bone strength and classification of fracture cases over traditional BMD measurements [17–19], motivating their integration into regulatory systems in the USA and Europe [20, 21]. In the context of exercise treatments for osteoporosis, current studies enabled quantifying the amount and distribution of hip strain for a variety of exercise types used for the prevention and management of osteoporosis [3, 10••, 22–24]. Hence, physics-based models can inform exercise prescription for osteoporosis.

Here, we review the literature on models of skeletal mechanics for informing exercise prescription in osteoporosis. We focus on the hip, which shows the smallest and most variable response to exercise of the anatomical regions susceptible to osteoporotic fracture [6] and, when fractured, carries the most severe health consequences [2]. The exercise type required for maximizing hip strength is identified using the weakest link concept in relation to studies of hip architecture and fracture mechanics. Current studies providing hip strain during a variety of exercise types are reviewed and discussed in the context of exercise recommendations for osteoporosis by ESSA [3]. Finally, we provide the authors’ perspective on the use of digital technology for enabling personalized prescription of exercise type and amount for optimal bone health.

## Exercise for Hip Strength

Bone strength is determined by the weakest link in the structure in relation to the specific mechanical environment, geometry and loading. Hip architecture displays asymmetric trabecular organization and cortical thickness adapted to support habitual physical activity. The principal compressive trabecular network transfers the compressive load generated at the hip joint to the thick (3–4 mm) medial femoral neck cortex. The principal tensile trabecular network runs through the medial femoral head, the thin superior neck cortex (< 1 mm) and the lateral greater trochanter, carrying the tensile load generated by bending of the neck. The structural asymmetry of the femur has been attributed to minimal loading in the superior neck cortex while walking due to the antagonist effect of the hip abductors, which generates compression for balancing the frontal plane moment, and the tension required to sustain the body weight [25]. The asymmetric structurally adapted hip displays approximately twice the strength under habitual loading compared with that associated with the impact from a sideways fall on the trochanter [26].

**Fig. 1:**
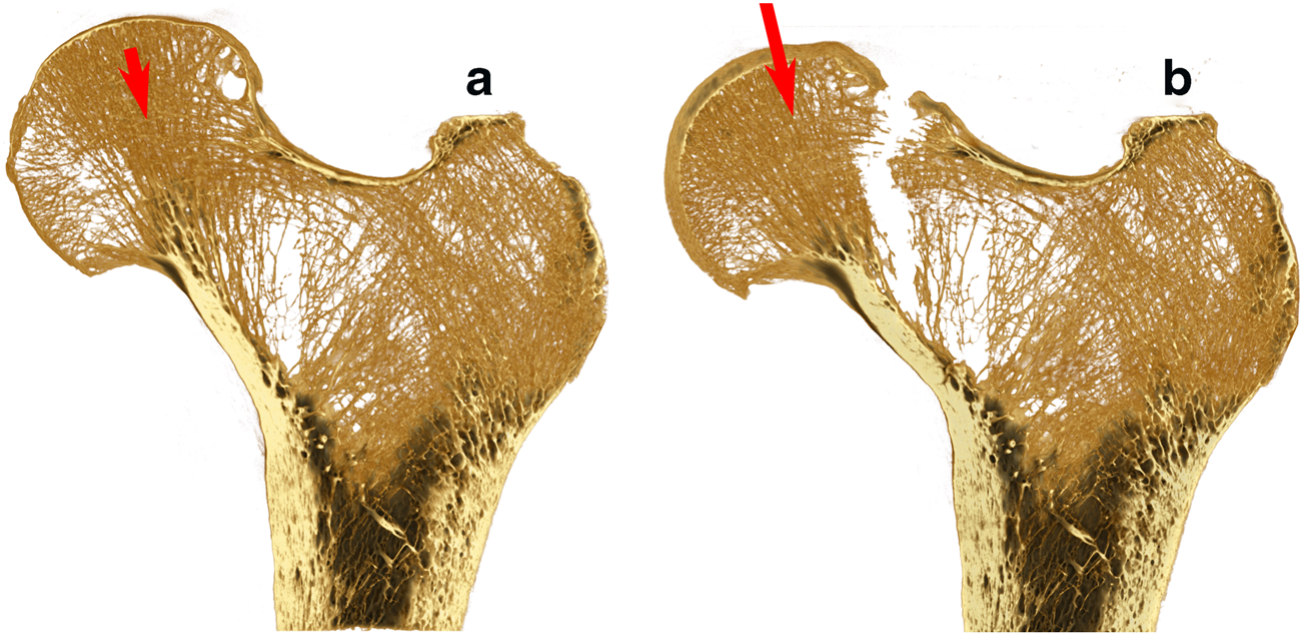
Cross-section images of a human femur (osteoporotic white women; age, 79 years; *T*-score = − 3) subjected to a single-leg stance configuration (red arrow) increased stepwise up to inducing a fracture opening in the superior sub-capital region of the neck. The figure displays the femur before load application (**a**) and after fracture occurred (**b**). Images were obtained using time-elapsed synchrotron-light micro-computed-tomography [30]

**Fig. 2:**
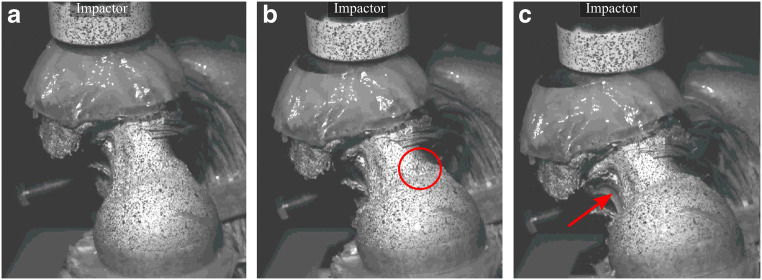
Three subsequent frames obtained using high speed cameras (at 15,000 fps) displaying fracture in an osteoporotic donor (white woman; age, 66; *T*-score = − 2.09) while replicating the impact resulting from a fall on a side (unpublished). Frames represent the femur before touchdown (**a**), at the time of the first cortical opening observed in the superior neck (**b**, red circle) and fracture progression to the medial neck (**c**, red arrow). Speckling and strain gauging seen in the images were part of a different study

Age-related bone loss causes diffused bone weakening and increased susceptibility to fracture under minimal load in low-energy falls, low dynamics events (e.g., stumbling) and spontaneously, for the weakest hips under intense muscle co-contraction [27]. Although a variety of fracture patterns have been observed, ranging from subcapital to subtrochanteric fractures, the thin superior neck cortex appears to represent the weakest link in the majority of fracture cases showing thinner superior neck cortices than non-fracture controls [28]. Laboratory experiments showed that the superior cortex opens in tension under habitual loading configuration [29] (Fig. 1) and crushes in compression while falling on the side (Fig. 2) in femurs of variable BMD [17], suggesting that osteoporosis affects strength but not the mode of failure of the hip. Therefore, it appears that promoting hip strength through exercise can be achieved by promoting either diffused bone accrual by increasing the strain intensity experienced during habitual activity or local thickening of the superior neck cortex by increasing bending of the neck. From an evolutionary perspective, promoting the thickening of the superior neck cortex through physical exercise means reversing the process of human evolution that made it thin [25].

## Classification of Studies of Hip Strain During Exercise

At the time of writing, searching Medline and PubMed using keywords hip, strain, exercise and model yields 51 studies published over the past 10 years, of which 11 used integrated models for providing information pertaining to hip strain in humans for a variety of exercise types [10••, 22–24, 31–37]. Integrated models combined musculoskeletal models of human motion providing muscle and joint forces during exercise [38, 39•, 40] and structural models of femoral mechanics providing femoral strain under prescribed muscle and joint forces. Studies have focussed on normal activities [10••, 22, 31, 33–37], impact and resistance exercises [10••, 22, 31, 36], age-related changes of hip mechanics during walking [37] and the effect of isolated hip-spanning muscle groups on hip mechanics [24]. Henceforth, such models are reviewed for providing guidance for designing exercise interventions targeting specific regions of the hip.

All the 11 studies analysed here assumed rigid skeletal and joint behaviour and optimal control assuming that muscles are recruited by targeting a prescribed function such as, for example, minimizing muscle activation or energy consumption. Optimal control was found to provide estimates of the hip contact force in agreement with published measurements [10••] and to exceed by less than 0.22 BW the hip contact force while walking obtained by imposing the model to follow (*R*^2^ = 0.60) some electromyography recordings in participants [13]. However, optimal control methods have difficulty in tracking recorded antagonist muscle contraction, which can drive the hip contact force up by multiples of the body weight during exercises requiring joint stabilization like stumbling [41] or reaction to unexpected motion perturbations [42]; these conditions were not considered by the studies analysed here. Regarding model anthropometry, most models scaled a generic body anthropometry to intersegmental lengths in participants, providing consistent cohort averages of the hip contact force (*R*^2^ = 0.81), femoral neck strain (*R*^2^ = 0.95; slope = 0.96–0.97) and a peak error equal to 4.2 BW [31]. Similarly, skin movements in common stereo-photogrammetry motion capture experiments were shown to provide consistent patterns and timing of the hip contact force but caused a variation of the force intensity equal to 1.8 BW [43]. For comparison, the hip contact force during walking is 2.5–4.5 BW [44] and over 9–12 BW can be expected in exercises for osteoporosis management generating 2–3 BW ground reaction force [3]. Therefore, the studies analysed here provided the main effect of exercise type while their validity in specific participants should be taken with caution.

Concerning the femur’s structural model, some studies modelled femoral strain using bone geometry and distribution in calibrated computed tomography (CT) images and a procedure validated earlier for calculating cortical strain (*R*^2^ = 0.89–0.95, [15, 16]) and hip strength (*R*^2^ = 0.80–0.85; [17]) [10••, 22–24, 32, 45]. The same procedure was used to model a osteoporotic donor (white women; age, 81 years old; *T* score = − 2.5) combining body anatomy from full-body CT imaging and dissection and a structural model of the femur experimentally validated (*R*^2^ = 0.95) later used for studying different exercises [22]. Other studies fitted a generic bone density map to a cohort reporting limited information on model validity [36, 37]. Therefore, it appears that validated models can be used to study the amount of strain under prescribed forces in participants while different technologies may enable ranking of exercise types. These differences should be considered when comparing different studies.

Models provided estimates of hip strain enabling ranking exercises consistently with their osteogenic capacity found in current randomized studies [46–49]. For example, 50 unilateral hops per day per 6 months caused 0.8–2.8% increase of femoral neck BMD in premenopausal women [46, 47] in agreement with higher strain while hopping and jumping over walking in all numerical studies analysed [10••, 22, 31, 36]. Squatting and deadlift exercises, as opposed to hip abduction exercises, caused concomitant strength increase of both the hip and the hip extensor muscles [48] in agreement with the ability of the hip extensor muscles, as opposed to the hip abductor muscles, to load the superior neck in our earlier numerical analyses [22–24]. Fast walking (6 km/h) was found to nearly double strain, either in tension or compression, experienced by the superior neck during normal walking (4 km/h) in agreement with earlier observation of diminished bone loss in a self-paced brisk walking exercise intervention [49]. Finally, the heterogeneity of the bone response to exercise observed by Lang and colleagues [48] is in agreement with the heterogeneity of hip strain reported by most numerical studies [10••, 22, 32].

Regarding model consistency, comparison between models is complicated by the limited number of studies analysed here, different procedures used for modelling femoral mechanics, often incomplete information of exercise kinematic and dynamic and by different mechanical variables for assessment. For example, different studies used principal strain components [36], effective strain [10••], strain energy density per unit of bone mass [22] and strain averages over different bone volumes [10••, 37]. To date, there is no gold standard for comprehensive verification of modelled lower limb muscle forces, except at the triceps surae [50] and quadriceps [51, 52], and limited verification data for hip contact forces [53] and femoral strain [54]. One study compared the tensile strain in the proximal lateral shaft during walking to corresponding strain measurement published for two hip-snapping syndrome patients (i.e., 1198–1454 με in tension and 393–948 με in compression) [54], hence providing a low-strain point for model verification [31]. A different high strain point for model verification may be obtained, for example, by comparing the strain calculated during high intensity safe exercises to bone damage threshold levels [22, 55]. We expect that the increase in the number of studies, the availability of benchmark data, open databases and cross-laboratory initiatives will soon promote consistency across and facilitate comparison between studies.

## Exercise Intensity and Type

The osteogenic response to exercise occurs at locations where strains exceed those of habitual loading, with the latter typically considered during normal walking in sedentary individuals (speed, 3.5–4.5 km/h). As such, exercise generating higher strain in the superior neck cortex than that during normal walking is preferable for promoting hip strength. During walking, the effective strain in postmenopausal women, averaged over 12 neck portions, in the anterior and superior femoral neck is 1045–1241 με [10••]. Gait speed is a well-known indicator of age-related functional decline [56]. Although Lim et al. showed that muscle contribution to acceleration of the centre of mass is invariant to age [57], Anderson and Madigan [37] showed that young participants (age, 25 ± 4 years) display higher ground reaction (9%), hip contact force (18%) and similar strain levels during late stance as compared to older counterparts (age, 79 ± 5 years) walking at the same speed. Since hip strain increased as BMD decreases, hip strain may also be an indicator of functional decline, independent from gait speed [37].

Other common activities including stair ambulation, fast walking and light jump in place confirmed minimal loading of the superior neck cortex theorized earlier by Lovejoy for upright locomotion [25] and display similar strain patterns [10••]. Nevertheless, stair ambulation was found to induce in the superior neck 30% higher effective strain (1410–1639 με) than walking, suggesting that stair ambulation may be a useful mechanical stimulus for the hip while ageing [10••]. Conversely, lower strain than walking was found during landing on both feet from a light jump in place generating 0.6 BW ground reaction force on each foot [10••]. In women aged over 60 years, the ground reaction force explained the 52–85% of the variance of the effective strain at mid-femoral neck [10••].

**Fig. 3:**
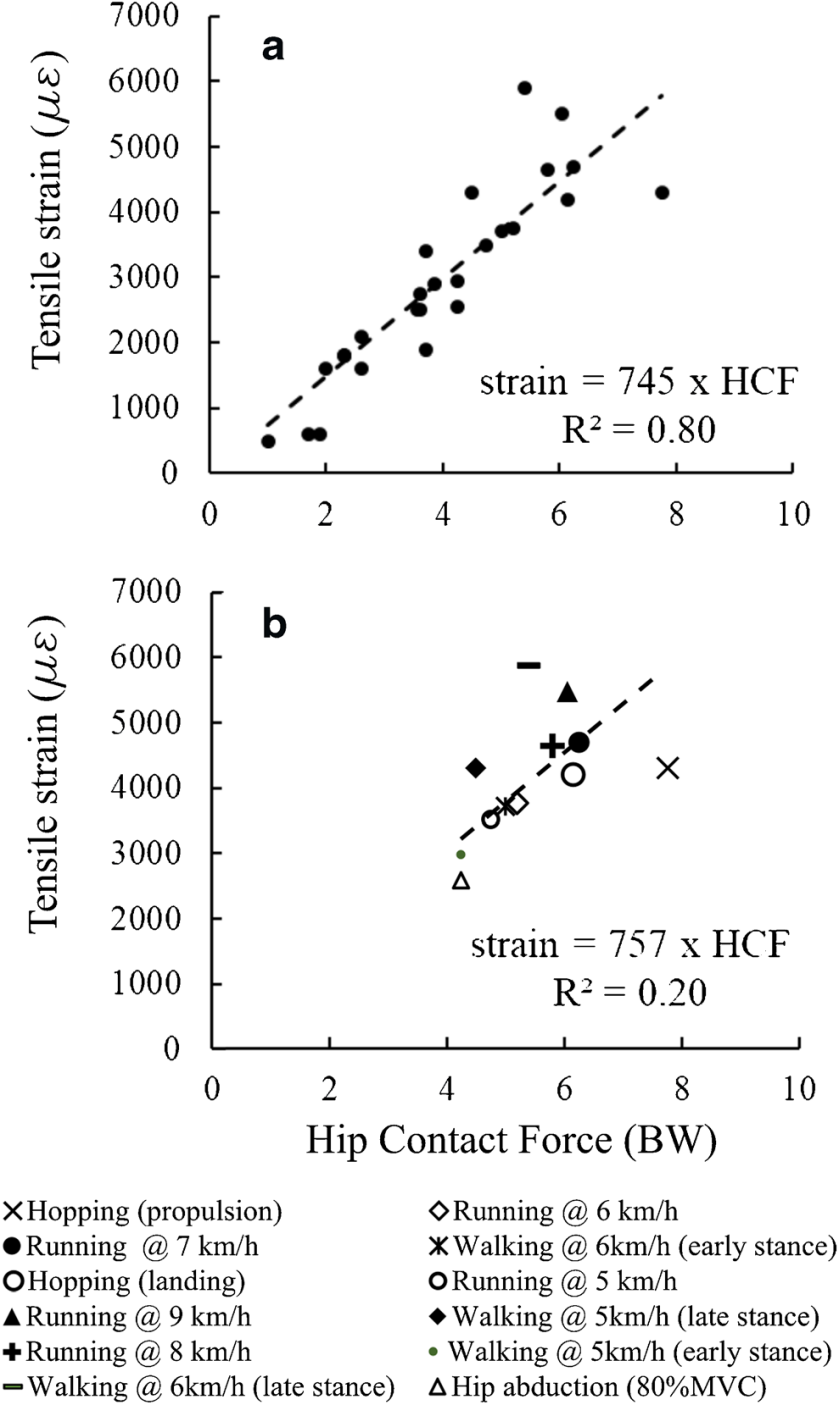
Linear regression analysis between the tensile strain in the superior neck and the intensity of hip contact force for the exercises analysed by Pellikaan and colleagues [35]. The regression was performed by pooling all the exercises together (Fig. 3a) and for the exercises inducing a hip contact force higher than that generated by normal walking (Fig. 3b)

Higher intensity exercises including fast walking, hopping, jumping and resistance training exercises about the hip were ranked using estimates of hip contact force and strain [36]. Hopping and running at 7–9 km/h generated the highest hip contact force (6–8 BW). Hopping generated about a threefold increase of the peak tensile strain in the inferior neck over every other exercise analysed and the highest compressive strain in the inferior neck. Fast walking (6 km/h) generated the highest tensile strain in the superior neck, closely followed by running (7–9 km/h), and the highest compressive strain, closely followed by hopping. Fast walking, hopping (propulsion) and running (9 km/h) induced 1.5–2.5 times higher strain than normal walking, either in tension or compression, in the superior neck. Fast walking and running at an increasing speed (5–9 km/h) caused similar strain levels in the superior neck, likely because of reduced muscle ability to generate support as speed increases [58]. All resistance exercises about the hip induced lower strain in the hip than normal walking. However, the hip torque generated during exercise was not provided. By pooling all the activities together, the hip contact force explained the 80% of the tensile strain variance in the superior neck. However, the strain variance explained by the ground reaction force decreased to 20% focusing on the 12 exercises generating higher hip contact force than normal walking (> 4 BW) (Fig. 3), complicating ranking of osteogenic activities using the hip contact force intensity and suggesting a specific effect of exercise type on the hip contact force direction.

The specific strain and strain energy generated per unit of ground reaction force and joint torque were studied using the model of an osteoporotic donor combining body anatomy from full-body CT imaging and dissection and a structural model of the femur experimentally validated (*R*^2^ = 0.95) [22]. The model was animated by weight-bearing activities recorded in a young body-matched volunteer and maximal isokinetic contractions about the hip and knee in a mixed cohort aged 61–78 years [59]. Long jumps generating 0.6–2.6 BW ground reaction force and maximal hip extension exercises caused approximately 5000 με tension in the superior neck and highly heterogeneous strain maps showing focal strain varying from the superior (hip extension and knee flexion) to the anterior neck (long jump). The specific strain energy generated per unit ground reaction force in weight-bearing exercises and per unit of joint torque in resistance training exercises varied by 2–10 times across exercises. Differences in the specific strain and strain energy across exercise were then attributed to the specific muscle contractions driving each different exercise.

**Fig. 4:**
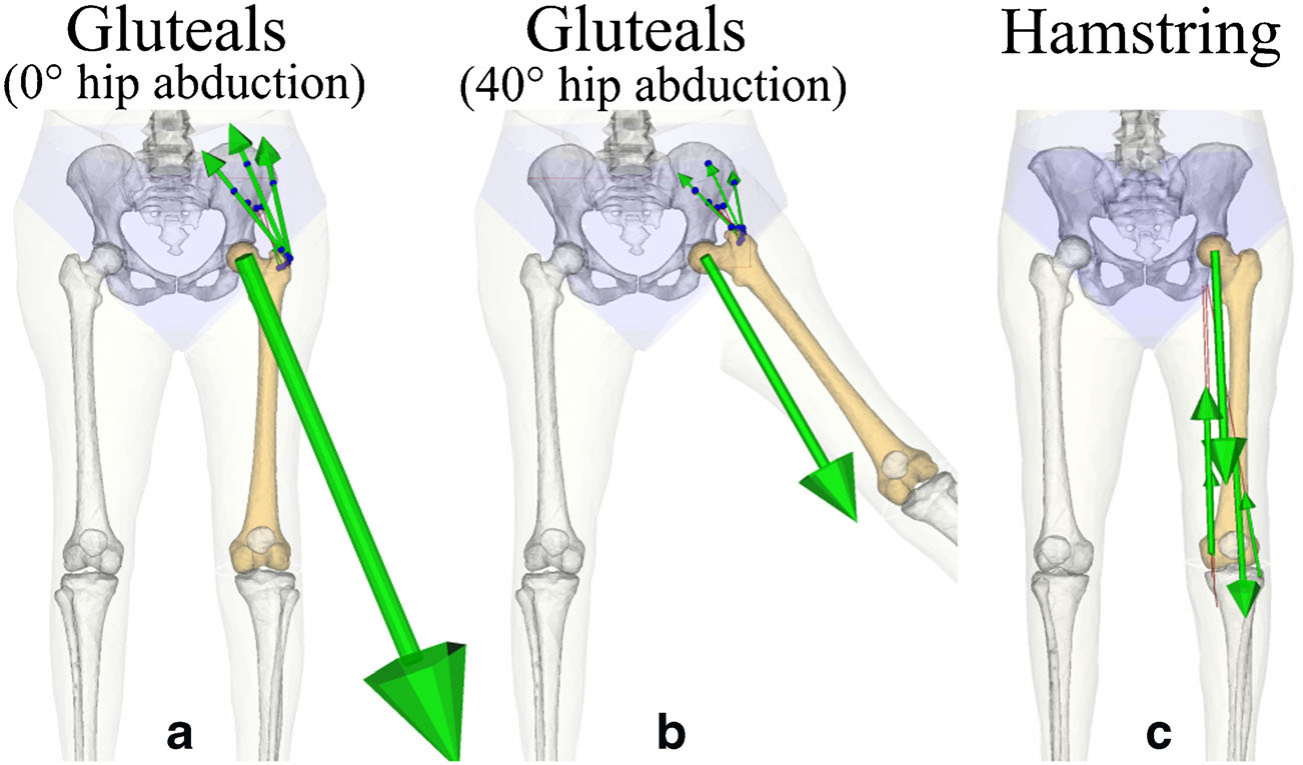
The hip load generated by contractions of the gluteus (gluteus medius and minimus) and of the hamstring muscles. The gluteus generates a mostly compressive load to the femoral neck at 0° hip abduction (**a**) and significant bending at 40° hip abduction (**b**). The hamstring generates a hip contact force at small angles from the diaphyseal axis causing substantial bending to the neck (**c**). Force vectors for the gluteus and the hamstring muscles are scaled differently to improve the readability of the figure. Animations representing the entire range of motion are available in the supplementary material

A study investigated the hip contact force orientation, intensity and hip strain potentially generated by each hip-spanning muscle group and their relation to anatomy and body posture [24] (Fig. 4). The hip abductor muscles generate predominantly compression at 0° hip abduction by pulling the greater trochanter toward the ilium and their force-generating capacity decreases as the hip abduction angle increases and loading of the neck becomes substantial bending at 40° hip abduction. The hip extensors (gluteus maximus and hamstring muscles) compress the femur between the pelvis and the tibia, generating a hip contact force at a small angle from the diaphyseal axis and causing mostly bending to the neck and tension in the superior neck. The iliopsoas, the primary hip flexor muscle, pulls the femur from the lesser trochanter compressing the posterior neck and tensioning the anterior neck. Interestingly, the hip contact force during normal daily activities, as measured in patients wearing a telemetric hip implant [44], was found to span a narrow central position in the envelope of the hip loadings potentially generated by each muscle separately [23], thereby supporting the use of multiaxial exercises to cause extreme non-habitual loading to the hip. This information can be used for determining the exercise type targeting specific hip regions.

## Exercise Recommendations

The exercise recommendations of the ESSA position statement include a mix of weight-bearing and resistance exercises of increasing intensity inducing from 2 to 3 BW ground reaction force for the management of osteoporosis to above 4 BW ground reaction forces for osteoporosis prevention [3]. Model results support ESSA’s current exercise recommendations and provide information for optimizing current exercise treatments.

Fast walking (6 km/h) and stair ambulation, not included in ESSA’s recommendation, may be easily integrated into normal daily activity to help mitigate the natural age-related bone decline. Running is unlikely to translate into increased mechanical stimulus of the hip compared with fast walking. High intensity jumping and hopping exercises may promote anabolic response of the superior neck. Multidirectional exercises may leverage the different capability of different muscle groups and postures for loading above usual levels different hip regions. Resistance exercises targeting hamstring and hip extensor muscles, among those recommended by ESSA, have the highest potential for loading the superior neck cortex [22]. Concerning exercise safety, exercises generating 2–3 BW ground reaction force in osteoporotic patients were found safe in a single validated model [22]. No study reported the safety of different exercise types in osteoporosis for a broader range of BMD levels.

Models can also contribute to the design of questionnaires of self-reported physical activity for relating exercise regimens to hip response and for guiding exercise recommendations. For example, the bone-specific physical activity questionnaire (BPAQ) combined self-reported physical activity and an osteogenic index, which was based on information on the ground reaction force, loading rate, frequency and resting time [60, 61]. Such questionnaire explained 65% of the variance of femoral neck BMD in young males [62, 63], improving the otherwise modest relationship of self-reported generic physical activity [64]. The specific strain and strain energy per unit of ground reaction force in the models can be used to weight exercises according to both their intensity (ground reaction force) and type (specific effect).

## A Perspective Toward Personalized Exercise Prescription

Personalized exercise prescription requires discerning individual features of the hip response to exercise that may be determined by complex interaction between anatomy, muscle function, bone distribution and metabolism. Therefore, accounting for the interaction between all these features can enable personalized prescription of exercise treatments for osteoporosis. Here, we provide the authors’ perspective toward personalized prescription of exercise-diet-drug treatments.

Significant progress has been made toward a fast, reliable and personalized physics-based model of human physiology. Current technologies can incorporate anatomical, mechanical, physiological and neurological features in each individual [40, 65]. The body anatomy in the model can be informed by medical imaging [66–68] or extracted from population databases [69, 70]. Statistical methods have been developed for improving the anatomical fidelity in the model using the limited set of information typically available in a clinical environment [71, 72]. Real-time or near real-time numerical methods have been developed to predict muscle and joint force [73], bone strains [74, 75] and strength [76]. Technologies have been developed to constrain the problem of muscle coordination to available observations of muscle activity [77, 78] and to reduce the bone position error in current motion capture technologies [79]. Similarly, bone anatomy and distribution can be obtained from calibrated CT images [10••, 22, 31] or extracted from population databases [76]. Access to high performance computing hardware, efficient new computational algorithms [74, 75, 80, 81] and open-source population databases [71] have reduced the burden of implementing robust models. These advances will enable personalized computational models of hip strength and strain during exercise.

Regarding the response of hip strength to exercise, recent evolutions of the mechanostat model proposed by H. Frost in 1983 describe how osteocytes and lining cells sense changes of the mechanical stimulus and adapt current bone structure by activating osteoblasts (i.e. bone-forming cells) and osteoclasts (i.e. bone-resorbing cells) [82–84] where the mechanical stimulus exceeds, or is lower, than its homeostatic value [85, 86]. Typically based on animal studies [87–89], these models can be used for determining the amount of bone deposited, or resorbed, as a function of exercise, individual metabolism, diet and pharmacological treatment [90, 91•, 92] for different anatomical locations and bone types [87–89]. In humans, bone adaptation theory has been used to relate normal activity to bone distribution [86, 93], for studying the long-term stability of total hip replacements [94–98] and the hip response to leg-press exercises [23]. However, most human studies focused only on the mechanical component of bone adaptation, but not metabolism [23, 86, 93–98]. Only one human study used models of bone metabolism focussing on changes of the mid-femoral diaphysis due to ageing, parathyroid hormone treatment and the lack of gravitational forces during space flights using a single representative mechanical loading [99]. Incorporation of models of hip metabolism and mechanics during exercise is the current focus of the bone research community.

In the coming years, it is likely to be possible to deploy a highly personalized computational model of hip strength and response to exercise with minimal imaging requirements in minutes, which can then be used to design the exercise regime for a given individual. The use of such models in combination with retrospective and longitudinal exercise interventions will allow appropriate identification of model assumptions and technology for personalized prescription of exercise type and amount for optimal hip strength.

## Conclusion

The analysis of the current studies on hip strain during exercise has shown that fast walking and stair ambulation, but not necessarily running, may help mitigating the natural age-related bone decline and may be easily integrated into routine daily activity. High intensity jumps and hopping promote hip strain in the superior neck higher than that during walking and multidirectional exercises may cause beneficial non-habitual strain patterns. Resistance knee flexion and hip extension exercises can induce high strain in the superior neck when performed using maximal resistance loadings in the average population. Information about exercise safety in the models analysed is marginal and at times contrasted likely due to limited model verification in some cases. Digital technologies can help study the interdependencies between anatomy, bone distribution, exercise, strain and metabolism thereby enabling personalized prescription exercise for optimal hip strength.

## Electronic Supplementary Material

ESM 1 (MP4 2988 kb)

ESM 2 (MP4 5823 kb)
